# Muscle Atrophy: From Bench to Bedside

**DOI:** 10.3390/ijms24087551

**Published:** 2023-04-20

**Authors:** Daniel Taillandier

**Affiliations:** UNH-Human Nutrition Unit-UMR1019, Clermont Auvergne University, INRAE-French National Research Institute for Agriculture, Food and Environment, F-63000 Clermont-Ferrand, France; daniel.taillandier@inrae.fr

The loss of muscle mass is a common adaptation to some physiological situations (e.g., bed rest), but also to many diseases (e.g., diabetes, cancer, heart failure, respiratory failure, renal failure and sepsis), which is due to an imbalance between protein synthesis and protein degradation [1]. Muscle loss contributes to the frailty syndrome and is associated with impaired quality of life and increased risk of death whatever the causal disease. In addition, muscle loss also decreases the efficiency of treatments, such as chemotherapy in cancer patients. Fighting against muscle loss is thus a major goal for ameliorating patients’ health. Compelling data demonstrate that increased proteolysis is often the main factor explaining muscle wasting [2]. However, muscle atrophy is a highly coordinated process that implies the concomitant regulation of anabolic and catabolic pathways, which suggests that we not restrict our studies to proteolysis. A potential strategy to improve patients’ condition is to reduce muscle wasting by regulating either proteolysis, protein synthesis or both. Interestingly, hibernating mammals are naturally resistant to muscle atrophy in conditions known to induce highly debilitating situations in humans, and more interestingly, this resistance can be transferred to other mammalian cells [3].

Depending on the catabolic situation, different signaling pathways controlling the main proteolytic pathways and protein synthesis can be implicated in the development of muscle atrophy, which implies a large panel of potential therapeutical targets for fighting against muscle atrophy [4]. Numerous studies in the past 20 years have identified key factors influencing skeletal muscle protein homeostasis in rodents [1]. There is no doubt that new strategies will be uncovered in the near future, and these may be pathology-specific or more widely applicable to several situations of muscle atrophy. However, there is still a long way to go before these strategies can be made applicable to patients.

The Special Issue of the *International Journal of Molecular Sciences* entitled “Muscle atrophy: from bench to bedside” includes a total of six contributions (three original articles and three reviews) providing new information in the field of skeletal muscle atrophy, with a special focus on key actors controlling muscle proteostasis.

Cussonneau et al. [5] addressed the role of transcription factor 4 (ATF4) in mice skeletal muscles undergoing hindlimb suspension (HS) atrophy and in muscles from hibernating brown bears, a naturally atrophy-resistant model. The activity of ATF4 was increased using a pharmacological drug (halofuginone) in mice and the authors found that ATF4-regulated atrogenes (atrophy-related genes) were upregulated in both HS mice and hibernating bears (note that ATF4 is upregulated is hibernating brown bears). Intriguingly, the up-regulation of the ATF4-regulated atrogenes was uncoupled from muscle atrophy in both animal models, and halofuginone treatment reproduced the muscle features of hibernating bears, with some notable atrophy resistance. The authors also found that halofuginone was able to mimic what happens in hibernating brown bears by differentially modulating actors of the TGF-β superfamily: the repression of transforming growth factor-β (TGF-β) and the activation of bone morphogenetic protein (BMP). This work paves the way for deeper investigation on the TGF-β/BMP balance, which may lead to the development of new strategies to fight against muscle atrophy.

Dowling et al. [6] addressed the potential use of markers for addressing the progressive loss of muscle mass and strength in elderlies, i.e., sarcopenia. The authors compiled proteomic data from elderlies and aged rodents and observed that the fiber shift (from fast to slow) appearing during aging may be a good readout for witnessing the appearance of sarcopenia. Likewise, key contractile proteins, such as myosin heavy chains, myosin light chains, actins, troponins and tropomyosins, appeared to be well-adapted to witness fiber switch linked to sarcopenia.

Valdebenito-Maturana et al. [7] addressed the mechanisms by which statins induce side effects such as skeletal muscle disorders including myalgia, myopathy and myositis. Indeed, statins are drugs widely used to reduce endogenous cholesterol synthesis, which in turn decreases cholesterol concentration, and thus, the risk of cardiovascular diseases. It is therefore highly important to understand the molecular mechanisms responsible for the side effects of statins. Here, the authors followed a particular track: the potential implication of transposable elements (TE) as potential mediators of statin. The focused on two statins (rosuvastatin and simvastatin) and found that several TEs were differentially expressed upon statin treatment. Interestingly, the number of TEs modulated was highly different depending on the statin, simvastatin being highly potent with the modulation of the expression of >1000 TEs. The authors identified several genes whose expression may be linked to muscle atrophy. Finally, the authors proposed a model in which epigenetic modifications in the TEs may increase their expression and subsequently downregulate their neighboring genes.

Van de Haterd et al. [8] reviewed the role of mitochondria in skeletal muscle atrophy during colorectal cancer cachexia (CRC). Mitochondria homeostasis plays an important role in muscle maintenance. Systemic inflammation is a hallmark of CRC and plays a crucial on skeletal muscle mitochondria. In particular, the IL-6-STAT3 pathway plays a pivotal role in skeletal muscle wasting by inducing skeletal muscle mitochondrial dysfunction. In CRC, an imbalance between mitochondria fission and fusion alters mitochondria homeostasis, thus resulting in a reduction in muscle oxidative capacity and an aggravated skeletal muscle atrophy. This mitochondria loss of function seems to be directly linked to an increased activity of the main proteolytic systems, with a cooperation between the UPS and the autophagy proteolytic systems, but the calpain system was also implicated. Mitochondria disruption led to apoptosis, but exercise (combined or not with EPO) was beneficial for both mitochondria biogenesis and function. Preventive exercise appears to be a promising approach for limiting CRC cachexia and future investigations will have to set up clinical training programs adaptable to patients’ exercise capacities. 

Winzer et al. [9] addressed the molecular and physiological alterations of the peripheral skeletal muscles in a rat model of heart failure (obese rats) with preserved ejection fraction (HFpEF). The main goal of this study was to investigate the potential beneficial effect of a sodium-glucose-transporter 2 inhibitor (Empagliflozin (Empa)) on skeletal muscle function and metabolism and mitochondrial function. Indeed, clinical studies provide evidence for the beneficial effect of such inhibitors by reducing the risk of cardiovascular death or hospitalization for HFpEF patients. Empa was effective for improving heart function in obese rats and Empa ameliorated skeletal muscle force with concomitant positive effects on mitochondrial function. 

Yoshihara et al. [10] provide an up-to-date review of sepsis-associated skeletal muscle wasting (SAMW), from the molecular mechanisms involved (mainly deciphered in rodent models) to the diagnostic and current treatments used in patients. Systemic inflammatory cytokines (e.g., IL-6, TNF-α, IFN-γ, and IL-1) are crucial actors of SAMW which occur through the activation of the three main proteolytic pathways: the ubiquitin proteasome system (UPS), the autophagy system and the calpains. Calpains are supposed to deconstruct the myofibrillar structure; autophagy is notably involved in the degradation of organelles such as mitochondria, while the UPS is directly involved in the degradation of contractile proteins and in the repression of protein synthesis, thanks to the expression of the muscle atrophy-related genes Atrogin-1 and MuRF-1. Several signaling pathways are involved in SAMW, including JAK/STAT, FoxO-Akt and NF-κB, all of them being directly implicated in the increased expression of proteolytic enzymes and the repression of the anabolic mTOR pathway. From a clinical point of view, the diagnosis of SAMW can be performed using several approaches (CT scan, MRI, functional tests, etc.), but few of them are usable routinely in a hospital setting, and future work may uncover plasma biomarkers (e.g., miRNAs). No pharmacological drug exists for fighting against SAMW, but the combination of mechanical stimulation (electrical muscular stimulation, physiotherapy and mobilization) and nutritional support helps to improve SAMW.
